# Virtual Reality in the Assessment, Understanding and Treatment of Mental Health Disorders

**DOI:** 10.3390/jcm9113434

**Published:** 2020-10-26

**Authors:** Giuseppe Riva, Silvia Serino

**Affiliations:** 1 Humane Technology Lab, Università Cattolica del Sacro Cuore, 20123 Milan, Italy; giuseppe.riva@unicatt.it; 2 Istituto Auxologico Italiano, IRCCS, U.O. di Neurologia e Neuroriabilitazione, Ospedale S. Giuseppe, 28824 Piancavallo, Italy; 3 MySpace Lab, Department of Clinical Neuroscience, University Hospital Lausanne (CHUV), 1011 Lausanne, Switzerland

**Keywords:** virtual reality, mental health, presence

## Abstract

Computer scientists usually describe virtual reality (VR) as a set of fancy hardware and software technologies. However, psychology and neuroscience are starting to consider VR as the most advanced form of human-computer interaction allowing individuals to act, communicate and become present in a computer-generated environment. In this view, the feeling of “being there” experienced during a VR experience can become a powerful tool for personal change: it offers a dynamic and social world where individuals can live and share a specific experience. For this reason, the use of VR in mental health shows promise: different researches support its clinical efficacy for conditions including anxiety disorders, stress-related disorders, obesity and eating disorders, pain management, addiction and schizophrenia. However, more research is needed to transform the promises of VR in a real clinical tool for mental health. This Special Issue aims to present the most recent advances in the mental health applications of VR, as well as their implications for future patient care.

Computer scientists usually describe virtual reality (VR) as a set of fancy hardware and software technologies [1]: a computer or mobile device (e.g., a smartphone) with a graphics card capable of interactive third-dimensional (3D) visualization, controllers, and a head-mounted display (HMD) embedding one or more trackers. This short description clearly identifies the core technological parts of a VR system [2]: input devices, output devices, and the virtual environment. Input devices comprise all the sensors and trackers that sense the individual’s actions (e.g., hand and head movements) allowing the user to interact with the virtual environment. Output devices include all the technologies (e.g., head-mounted displays or CAVEs) that provide continuous computer-generated information to the user. Finally, the virtual environment (VE) is the 3D simulated scenario generated by the computer or the mobile device. VEs are designed to be explored, so users can interact (e.g., moving, pushing, picking, rotating, etc.) with their contents. In multi-user virtual environments (MUVEs) two or more users can share the same simulated scenario and communicate and/or interact inside it. To allow communication and interaction between users, MUVEs use avatars—personalized graphical representations of the individuals—that are directly controlled by their movements in real time. Embodied virtual agents, on the other hand, are graphical representations of the individuals controlled by the computer itself using an artificial intelligence program.

However, psychology and neuroscience are starting to consider VR as the most advanced form of human-computer interaction because it allows individuals to act, communicate and become present in a computer-generated environment [3,4]. In this view, the feeling of “being there” experienced during a VR experience can become a powerful tool for personal change [5,6]: it offers a dynamic and social world where individuals can live and share a specific experience. For this reason, the use of VR in mental health shows promise: different researches support its clinical efficacy for conditions including anxiety disorders, stress-related disorders, obesity and eating disorders, pain management, addiction and schizophrenia [7,8,9]. However, the use of VR in clinical practice has long been limited by two main factors: the lack of usability and the cost of virtual tools [10,11].

The first generation of VR devices, available between 1990 and 2015, was characterized by a series of significant shortcomings - low display resolution, limited field of view, and uncomfortable designs—producing different side effects to their users. In particular, motion sickness (due to low display quality) and neck pains (due to the weight of the HMD), limited the use of this technology in a clinical setting. Moreover, the first VR systems required expensive HMDs paired with equally expensive high-end computers usually costing 20,000/50,000 USD. Finally, developing and using a VR experience required a significant technical knowledge that was typically unavailable in hospitals and/or clinical centers.

2016 saw the release of the first generation of HMDs targeted at consumers. The Oculus Rift—an HMD developed and manufactured by Oculus VR, a division of Facebook Inc., and sold for 600 USD—marked a new generation of VR devices (see Table 1) that is revolutionizing how VR is used in general. In a few years, the cost of a complete VR device—including input, output, and 3D graphic computation—dropped by tens of thousands of dollars to just a few hundred, the price of the cheapest standalone VR systems [12]. However, more research is needed to transform the promises of VR in a real clinical tool for mental health. This Special Issue aims to present the most recent advances in the mental health applications of VR, as well as their implications for future patient care.

In most cases, VR is used as a “simulative tool” to recreate ecologically valid scenarios reproducing feared/critical situations (i.e., fear of speaking in front of an audience) with a precise control over the stimuli delivery according both to therapeutic strategies and patients’ needs [3,4]. In this way, patients can enter simulations of the situations that are extremely dangerous to experience in real-life (i.e., fear of heights), thus extending the boundaries of existing treatments. Moreover, VR permits the repeated delivery of the same scenarios while allowing the anxiety to attenuate, graded in difficulty and customized for each specific patient. VR-based treatments including simulations have been successfully implemented and tested in different clinical trials, especially for treating anxiety disorders [13,14,15,16,17], stress-related disorders [18], phobias [19], and post-traumatic stress disorders [20,21].

In order to further explore the potential of VR in the exposure therapy for anxiety disorders, in the current Special Issue Guitard et al. [22] compared the traditional exposure using imagination through a personalized catastrophic scenario to exposure in VR with a standardized scenario in 28 patients suffering from generalized anxiety disorder (GAD). In both cases, a neutral scenario served as a baseline. Their results indicated that the standardized virtual scenario induced higher anxiety when compared to the neutral one, thus suggesting the possibility of using it for the treatment of patients suffering from GAD.

In the same vein, Peñate et al. [23] tested whether the exposure to virtual phobic stimuli in a group of 32 patients with specific phobias (i.e., small animals) would activate the same brain regions as the exposure to real image stimuli. A specific inspection of the amygdala and insula detected higher activations in response to real images, but those areas have been activated also in response to virtual stimuli. Globally, these findings gave additional support for the use of virtual stimuli in cue-exposure treatments for phobias.

Moreover, Montana et al. [24] critically reviewed 11 VR-based trainings for the enhancement of emotion regulation strategies and wellbeing in young and older adults. Included trainings covered different techniques, such as the Mindfulness-Based Stress Reduction protocol, the biofeedback and neurofeedback trainings as methods for the regulation of physiological arousal, and the promotion of outdoor/indoor activities for the enhancement of subjective wellbeing. Globally, the use of VR in this research field is promising since it allows individuals to learn emotion regulation strategies in the context of life-like virtual environments.

Finally, Lopez-Valverde et al. [25] critically reviewed 31 studies and provided additional evidence to the idea that VR can be used as an effective distraction method able reduce pain and anxiety in patients undergoing a variety of dental treatments.

An expanding area of research is the exploitation of VR for delivering cognitive assessment and trainings in neurological conditions, i.e., patients suffering from neurodegenerative diseases or post-stroke individuals. VR has a prominent place among other advanced technologies offering crucial advantages for implementing cognitive trainings [26,27,28,29,30]. Patients can perform cognitive exercises in controlled and secure environments, where it is possible to calibrate the difficulty/intensity of the tasks according to specific patients’ needs. Virtual exercises mimic real-world tasks (i.e., ecological validity) and they offer greater chances for transferring learnt abilities to real life [31]. These environments offer also a multisensory stimulation (i.e., vision, touch, motor), with a fruitful rebound on brain plasticity [27]. Several systematic reviews and meta-analyses indicated the efficacy VR for cognitive interventions for individuals with cognitive impairments and Alzheimer’s Disease [32,33], for alleviating motor and non-motor symptoms of Parkinson’s Disease [34], or for improving spatial neglect (i.e., failure respond to contralateral stimuli) in post-stroke individuals [35].

In this perspective, in this Special Issue, Tuena et al. [36] and Montana et al. [37] focused their efforts in reviewing experimental studies about the use of VR as an instrument for implementing navigational assessment tasks and trainings. Tuena et al. [36] systematically reviewed 31 experimental studies about the so-called “virtual enactment effect”, thus providing further support about the “embodied” potential of VR. Indeed, Brooks and colleagues [38] pioneered the idea that the active navigation in virtual environments by means of input tools could be considered as an analogous to the “enactment effect” thanks to the subsequent enhancement of memory performances by motor information. This systematic review shaded light on the key role of the sensorimotor systems for spatial and episodic memory assessment and rehabilitation by emphasizing the crucial role played by VR in future cognitive interventions. Montana and colleagues [37] critically reviewed 16 VR-based trainings for patients with spatial memory disorders (such as, patients with mild cognitive impairment, Alzheimer’s disease, traumatic brain injury, multiple sclerosis, stroke, etc.). Although further studies are needed to strengthen the methodological quality of studies in this research area, promising results about the possibility of using VR to deliver training to improve navigation and orientation abilities in several disorders were discussed. In a similar vein, Bevilacqua et al. [39] focused their work on reviewing existing randomized controlled trials in the field of VR-based training for geriatric population, considering only non-immersive systems. The idea is that non-immersive systems have shown to be highly accepted by older people. They critically reviewed 11 trials, thus suggesting that the application of VR has globally a positive impact on the rehabilitation of the most predominant geriatric syndromes.

In the same research domain, Thapa et al. [40] demonstrated the efficacy of an immersive VR-based cognitive training in a sample of 68 individuals with mild cognitive impairment. The proposed VR-based training (three times/week, 100 min each session) included four types of VR game-based contents to improve different cognitive functions (i.e., attention, memory and processing speed). Results indicated that the experimental group exhibited a significant improvement in executive functions, in the gait speed and in the mobility after the training. Finally, Cabinio et al. [41] tested the potential of a novel smart aging serious game (SASG) in discriminating older adults suffering from amnestic mild cognitive impairment from cognitively healthy controls. Results showed similar discriminating abilities for SASG and gold standard tests, and a greater discrimination ability compared to non-specific neuropsychological tests.

As concern the use of VR as “assessment tool”, Alcañiz Raya et al. [42] investigated the possibility of discriminating children with autism spectrum disorder (ASD) from typically developed children through body movements’ data analysis in a real-simulated imitation task implemented in VR. Their results indicated the feasibility of applying machine learning methods and VR-based ecological tasks to identify body movements’ biomarkers that could contribute to improving ASD diagnosis.

In the same vein, Pedroli et al. [43] tested 29 patients with obsessive-compulsive disorder (OCD) and 29 controls with traditional neuropsychological tests and a validated protocol implemented in VR (virtual multiple errands test—VMET) for the assessment of executive functions. They demonstrated the possibility of discriminating OCD patients from controls using scores from three neuropsychological tests and two indices from VMET, opening a novel scenario for future protocols based on VR-based assessment tool and computational techniques.

In the last 40 years, VR has offered innovative solutions also for assessing and treating body representation disturbances in eating and weight-disorders [4,44,45]. One of the most recent trends for treating body representation disturbances in patients suffering from anorexia nervosa (AN) is the exploitation of the so-called VR-based body ownership illusions [46,47,48]. Advances in technology permits the experimental induction of embodying an entire virtual body thanks to a multisensory stimulation experienced both on own real body and on the fake one in a synchronous way.

In this Special Issue, Porras Garcia et al. [49], Scarpina et al. [50], Provenzano et al. [51] and Matamala-Gomez et al. [52] induced in participants the feeling of being the owner of another fake virtual body and investigated the impact of such illusion in modulating body percept. Porras Garcia et al. [49] introduced some variations to the traditional illusion to further increase its illusory strength; they applied the synchronous multisensory stimulation on the entire participants’ body, and they created a real-size virtual body, based on each participants’ silhouette. They found that participants showed higher levels of body concerns and body anxiety after owning the larger-size body, than after owning the real-size one. The synchronous visuo-tactile group had higher scores in comparison to the asynchronous one, although the differences did not reach statistical significance. Scarpina et al. [50] employed the traditional virtual body ownership illusion in 15 participants with obesity and in a sample of normal-weight participants. Results indicated that participants with obesity as well as controls successfully experienced the illusion; both groups reported a decrease in the estimation of the abdomen’s circumference (congruently with avatar size) after the synchronous condition. Globally, these studies encouraged further research exploiting the use of VR as an “embodied tool” for modulating body representations in clinical populations, specifically in terms of its potential therapeutic use [46].

In addition, Provenzano et al. [51] applied the illusion with some modifications: they individualized the avatar according to participants’ bodies and the evaluated the effect of the embodiment strength using both explicit (i.e., the perceptual and emotional aspects of body image distortion) and implicit (i.e., body temperature) measures. They found that the embodiment was stronger after synchronous stimulation for both groups, but it did not reduce body image distortions in patients with AN. They found that the illusion succeeded in eliciting affective reactions: patients with AN experienced more negative emotions after embodying the fattest avatar.

VR-based bodily illusions are useful also for modulating the pain threshold in healthy subjects, with potential therapeutic implications for individuals with chronic pain. Matamala-Gomez et al. [52] aimed at deeper understanding the mechanisms of visual distortion on pain response in 27 healthy participants; in particular, they reproduced the telescoping effect in amputees (i.e., phantom limbs can be perceived as gradually retracting inside the stump). Results indicated that embodying a distorted virtual arm increased an autonomic response (i.e., skin conductance response) to a threatening event (i.e., virtual needle) with respect to embodying a normal control arm, but when participants embodied a reddened-distorted virtual arm, the skin conductance response was comparatively reduced in response to the threat. These findings provided further support to the use of VR for the modulation of pain responses by systematically changing the representation of the telescoped limb, with crucial implications for its use in integrated treatments to reduce chronic pain in clinical population.

Finally, a recent Cochrane review indicated that VR could play a role to provide schizophrenic patients with customized interventions able to increase their compliance by enhancing their cognitive skills [53]. Findings from studies carried out so far are promising, but there is an urgent need of high-quality studies.

In this domain, in this Special Issue, Drori et al. [54] presented and tested a novel VR paradigm to investigate the sense of reality (SoR), i.e., the ability to discriminate between true and false perceptions, that is a central criterion in the assessment of neurological and psychiatric health. They induced hallucination-like visual experiences and tested their effects using both objective and subjective measures of SoR. Results indicated a novel psychophysical link between the sensitivity to alterations of reality and prodromal psychotic symptoms.

In the same perspective, Stern et al. [55] focused their attention on one aspect of the bodily self, namely the sense of agency (i.e., the feeling of controlling our body’s actions). Starting from literature suggesting that the sense of agency is impaired in psychosis, with stress influencing this relationship, they found that an increased alteration of the virtual hand was significantly associated with a decrease in subjective ratings of sense of agency and body ownership. Moreover, they found that sense of agency was not related to the trait anxiety neither to induced stress.

In conclusion, while VR applications such as those discussed in the articles above are addressing very specific ways of supporting mental health, we believe that a new era in computing, one that aims directly at improving people’s psychological mental health will only be possible when we will able to exploit all the potential of this exciting technology. More, the design goal of achieving VR experiences for assessment and treatment in mental health requires an interdisciplinary approach, integrating knowledge and ideas from disciplines such as computer graphic, neuroscience, social and cognitive psychology, multi-sensory perception, cognition, multimedia development, and healthcare. In order to build VR tools which can effectively improve our approach to mental health, it will be necessary to incorporate and integrate ongoing insights from these fields into next-generation research exploiting all the dimensions of VR. In fact, VR is at the same time a simulative technology, a cognitive technology, and an embodied technology [3]. These features will make VR the perfect tool for experiential assessment and learning with great clinical potential. We hope that this new breed of artifacts will build on the work described in this special issue.

## Figures and Tables

**Table 1:** Commercially available VR devices.

	PC Based				Mobile Based			Console Based	Standalone		
Mobility Required											
**System**	Oculus Rift S	HTC Cosmos/	Valve Index	HP Reverb G2	Samsung Gear VR	Google Cardboard	Google Daydream	PlayStation VR	Xiaomi MI VR	Oculus Quest 2	Lenovo VR Classroom 2
		Vive Pro/									
		Pro Eye									
**Cost (USD)**	399	699/	99	599	99	10–50	69–149	299	199	299	399
		1199/									
		1599									
**Hardware Requirements**	High-End PC (>1000 USD)	High-End PC (>1000 USD)	High-End PC (>1000 USD)	High-End PC (>1000 USD)	High-End Samsung Phone (>600 USD)	Middle/Highend Android phone or iPhone (>299 USD)	High-End Android Phone (>499 USD)	PS4 (299 USD) or PS4 Pro (399 USD)	None	None	None
									(Interna l Snapdragon 821 processor)	(Internal Snapdragon XR2 processor)	(Internal Snapdrag on 835 processor)
**Resolution**	2560 × 1440	2880 × 1660	2880 × 1660	2160 × 2160 (per eye)	2560 × 1440	Depends on the phone (minimum 1024 × 768)	Depends on the phone (minimum 1920 × 1080)	1920 × 1080	2560 × 1440	2560 × 1920 (per eye)	2160 × 1920
**Refresh Rate**	80 Hz	90 Hz	120/144Hz	90 Hz	60 Hz	60 Hz	90 Hz minimum	120 Hz	72 Hz	90 Hz	75 Hz
**Field of View**	115 degrees	110 degrees	130 degrees	114 degrees	101 degrees	from 70 degrees	96 degrees	100 degrees	90 degrees	100 degrees	110 degrees
**Body Tracking**	*High*: head tracking (rotation) and volumetric tracking (full room size—15 × 15 ft—movement)	*High*: head tracking (rotation) and volumetric tracking (full room size—15 × 15 ft—movement)	*High*: head tracking (rotation) and volumetric tracking (full room size—15 × 15 ft—movement)	*High*: head tracking (rotation) and volumetric tracking (full room size—15 ×15 ft—movement)	*Medium*: head tracking (rotation)	*Medium*: head tracking (rotation)	*Medium*: head tracking (rotation)	*Medium/High*: head tracking (rotation) and positional tracking (forward/backward)	*Medium*: head tracking (rotation)	*Medium/High*: head tracking (rotation) and volumetric tracking (full room size—15 × 15 ft—movement)	*Medium/High*: head tracking (rotation)
**User Interaction with VR**	*High* (using controllers)	*Very High* (using controllers and eye tracking)	*High* (using controllers)	*High* (using controllers)	Medium (using gaze, a built-in pad or joystick)	*Low* (using gaze or a button)	*Medium* (using gaze or a joystick)	*High* (using a joystick or controllers)	*Medium* (using gaze, a built-in pad or joystick)	*High* (using controllers or hand tracking)	*Medium* (using gaze, a built-in pad or joystick)
**Software Availability**	Oculus Store	Steam Store	Steam Store	Steam Store and Windows Mixed Reality Store	Oculus Store	Google Play or IOS Store	Google Play	PlayStation Store	Oculus Store	Oculus Store	Google Play and Lenovo ThinkReality

VR: virtual reality; PC: Personal Computer
